# Automated tracking and analysis of ant trajectories shows variation in forager exploration

**DOI:** 10.1038/s41598-019-49655-3

**Published:** 2019-09-13

**Authors:** Natalie Imirzian, Yizhe Zhang, Christoph Kurze, Raquel G. Loreto, Danny Z. Chen, David P. Hughes

**Affiliations:** 1 Department of Entomology, Pennsylvania State University, University Park, PA USA; 2 Department of Computer Science & Engineering, University of Notre Dame, Notre Dame, IN USA; 3 Department of Biology, Pennsylvania State University, University Park, PA USA; 4 Department of Entomology, Federal University of Viçosa, Viçosa, Minas Gerais Brazil

**Keywords:** Behavioural methods, Behavioural methods, Machine learning, Behavioural ecology, Behavioural ecology

## Abstract

Determining how ant colonies optimize foraging while mitigating pathogen and predator risks provides insight into how the ants have achieved ecological success. Ants must respond to changing resource conditions, but exploration comes at a cost of higher potential exposure to threats. Fungal infected cadavers surround the main foraging trails of the carpenter ant *Camponotus rufipes*, offering a system to study how foragers behave given the persistent occurrence of disease threats. Studies on social insect foraging behavior typically require many hours of human labor due to the high density of individuals. To overcome this, we developed deep learning based computer vision algorithms to track foraging ants, frame-by-frame, from video footage shot under the natural conditions of a tropical forest floor at night. We found that most foragers walk in straight lines overlapping the same areas as other ants, but there is a subset of foragers with greater exploration. Consistency in walking behavior may protect most ants from infection, while foragers that explore unique portions of the trail may be more likely to encounter fungal spores implying a trade-off between resource discovery and risk avoidance.

## Introduction

Resource acquisition drives animals into new territories, while threat avoidance limits where animals move. A consistent threat is the presence of infectious propagules of parasites and these are hypothesized to be major determinants of the distribution of animals in the wild^1^. Examples of animals avoiding pathogen contaminated areas span diverse taxa, from mammals to insects, implying anti-parasite behavior is widespread^1–5^. Central place foragers are interesting in the context of parasite avoidance as they must obtain food while avoiding threats with the additional constraint of returning to a defined location after each trip. For volant central place foragers, like wasps, bees, bats and birds, much of the trip is through the air likely reducing contact with infectious material. However, for taxa which walk on the ground (e.g. ants), encounters with parasite propagules are presumably higher^6^. Unlike threats from mobile predators and competitors, parasites could directly alter movement patterns since infection occurs from a stable location on the ground. For social organisms, it would be advantageous to avoid pathogen contaminated areas in order to protect the entire colony from becoming infected.

While some ant species send workers out from the colony to forage independently, other ant species use highly coordinated groups to forage, often facilitated through chemical signaling^7^. Group foraging via chemical trails can lead to semi-permanent trails known as ‘trunk trails’^8^. Trunk trails stimulate research interest largely from the perspective of the self-organization behavior of ants, such as how ants regulate traffic^9–11^. Trunk trails have also been studied from the perspective of their temporal and spatial dynamics as well as their energetic value in terms of efforts expended and resources obtained^12,13^. Yet, studies have not investigated how utilizing the same trails day after day impacts the exposure of ants to threats. Moreover, studies on ant foraging have largely occurred in a laboratory setting, and of the work that took place in the field, most studies relied on human observation or manipulated the environment in some way (see references in Supplementary Table S1). An ant species that forages collectively and predictably in time and space would be useful to assess the relationship between trail behavior and risk avoidance.

A potential system is the carpenter ant *Camponotus rufipes* in southeastern Brazil, which forms trunk trails lasting for multiple months^14,15^. Colonies of this ant were recorded as having a chronic infection by the fungal parasite *Ophiocordyceps camponoti-rufipides* across 20 months^16,17^. This fungus manipulates foragers to leave the nest and die biting the underside of a leaf^17,18^. To complete its lifecycle, the fungus must grow out of the ant cadaver and form a fruiting body that releases spores onto the ground below that will infect other ants^18^. Cadavers are found attached to leaves surrounding the ant nest^17^. The chronic nature of infection at the colony level means the spores of the pathogen are continuously in the environment from the perspective of the foragers. The spores are curved and large (80–95 microns)^16^ implying they do not travel far and land on the nearby trails once released from ant cadavers that hang above trails. Spores germinate to produce infectious secondary spores on hairs (capilliconidia) which attach to ants as they walk over them^19^. Thus, infection does not require a spore to hit an ant as it walks on a trail below a cadaver. Instead, the trail substrate itself serves as the source of contamination.

Foragers of the carpenter ant *C*. *rufipes* mostly collect nectar from hemipteran secretions and extrafloral sources^14,20^. The exploitation of a stable resource suggests that the most efficient way for a colony to obtain resources is for the majority of foragers to walk directly to the food source, utilizing trails near the colony entrance as a highway. However, if all foragers walked directly towards the food source, this would hinder the colony’s response to changes in resource availability. We hypothesize that some individual trajectories will show evidence of searching behavior, but the majority of ants will walk directly across the trail and cover similar areas limiting the exposure of most ants to threats.

We studied the trails of seven *C*. *rufipes* colonies in their rainforest habitat to determine how individual ant trajectories vary in their consistency and coverage of trail space to investigate whether all foragers are at equal risk of encountering a fungal spore. Importantly, we studied ant movement on undisturbed trails, keeping pathogen risk at natural levels and including the factors undetectable to humans that influence ant foraging. We devised a system of recording trails using infrared lights and modified cameras to contend with the nocturnal foraging of this species. We then used computer vision and deep learning to automate ant tracking then characterized forager trajectories on speed, straightness, direction, and exploration.

First, we focused on the straightness of trajectories to assess the efficiency of the colony in food retrieval and to investigate whether some ants are engaged in searching behavior. Next, we analyzed the tendency of trajectories to cover unique areas of the trail through calculation of an “exploration index” of each trajectory. We predicted that most trajectories will have high straightness and low exploration scores as this increases food retrieval while limiting risk exposure. We then investigated the relationship between straightness and exploration, as well as exploration and time. We predicted that ants that walk straight across the trail are more likely to cover the same area of the trail as other ants (low exploration), while ants with lower straightness scores are more likely to walk over a new area of the trail (high exploration). We also predicted exploration levels would be higher at the beginning of a foraging period, as this is when the pheromone trail would be the weakest. We found that some ants wander when crossing the trail and these ants are more likely to explore a unique area of the trail, possibly increasing the flexibility of the foraging system by heightening food discovery. Conversely, covering a new area of the trail could expose wandering ants to threats other ants may avoid through following the main foraging trail.

## Methods

### Study site

Fieldwork took place at the Research Station of Mata do Paraíso, Universidade Federal de Viçosa, Minas Gerais, Southeast Brazil (20°48′08S 42°52′31W) between 10 and 25 January 2017. The carpenter ant *Camponotus rufipes* is abundant in this area, forming trails lasting multiple months^14,15^. The forest floor in the area of study is usually covered in 10–20 cm of leaf litter. Instead of traversing through the leaf litter, *C*. *rufipes* trails often use ‘bridges’ composed of woody debris, lianas, and tree branches 2 cm or more above the leaf litter^15^. Occasionally, when there are patches of clear soil (usually due to human made paths) trails would cross these areas. Ants forage at night and activity peaks in the early evening.

### Trail filming

Trails from seven different *C*. *rufipes* nests were filmed between 10 and 25 January 2017. Nests were selected based on their location and structure. Only nests found above the ground with nest material clearly visible were used. Trails were filmed before a branching point from the main trail so that ants were filmed coming directly from or towards the nest. In the case where multiple trails came from one nest, the busiest trails were selected. The width of the branches filmed ranged from 0.8 cm to 7 cm (mean ± standard deviation; 2.97 cm ± 2.53) and the length of the area filmed for all branches was approximately 15 cm.

**Figure 1:**
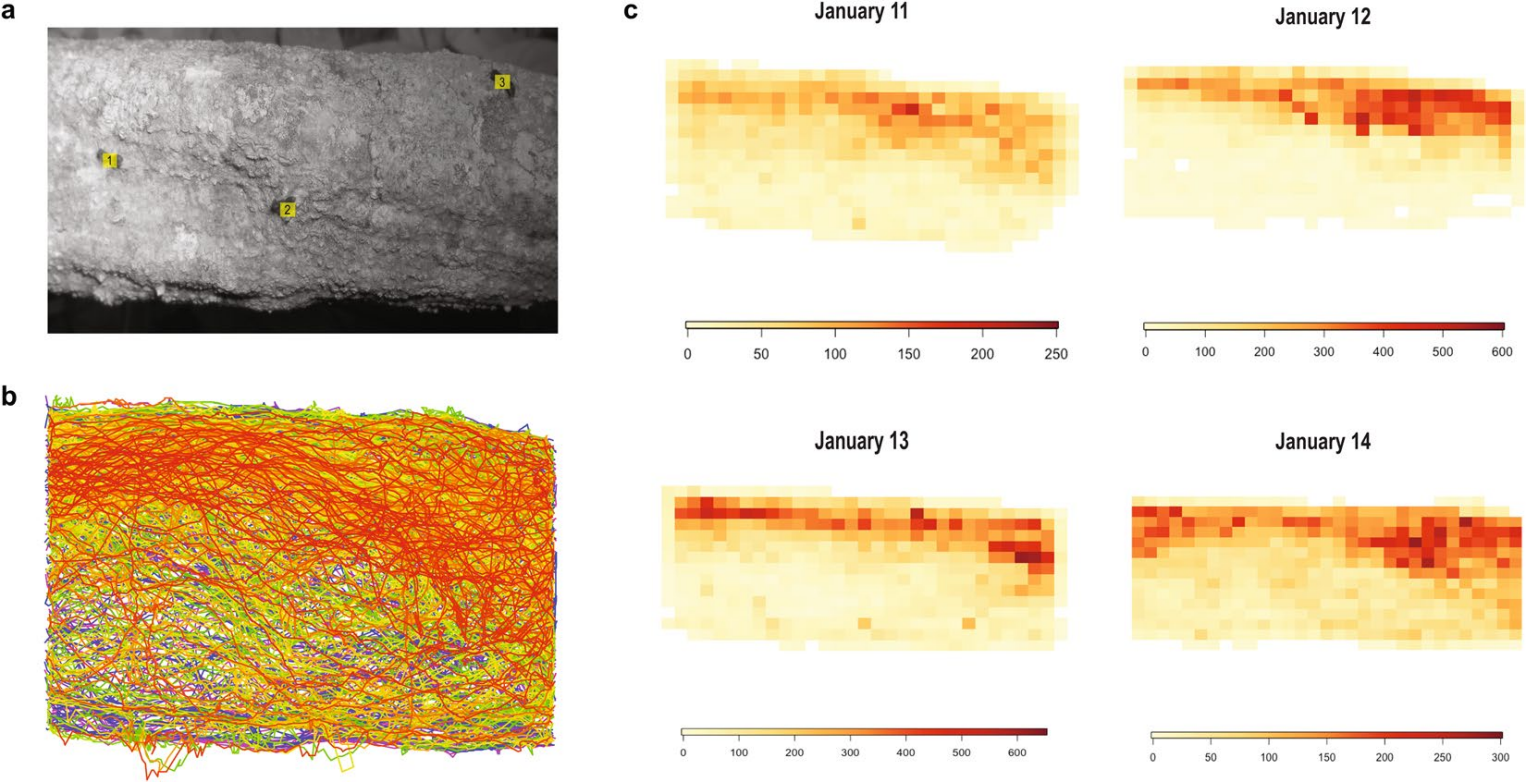
Trail image, trajectory overlay, and collective movement pattern. (**a**) Example trail image from GoPro footage of colony MP1. Individual ants are labeled with identification numbers. (**b**) All of the trajectories from a single night of footage (January 14) at colony MP1. Each line across the trail represents a different ant, with the different colors distinguishing between different ant tracks. (**c**) The trail space from (**a**) was divided into a grid with each square representing approximately 1 cm^2^. The number of times an ant walks into a square of the grid was calculated and the darker colors represent areas of the trail that ants walked over more. Each heatmap represents a different date (January 11 through January 14) from approximately the middle of the night to control for differences in the timing of filming. Different scales were used for each night, due to variance in the number of ants that walked across the trail.

GoPro cameras (model: HERO 3+, GoPro, Inc., San Mateo, USA) with a modified infrared filter (RageCams.com, Michigan, USA) were used for filming. Stakes were placed 30 centimeters from the trails and 30 cm medium trigger clamps (DWHT83140, DeWalt, Towson, USA) were attached to the stakes. Cameras were attached to clamps so that cameras were approximately 30 centimeters above the trails looking down at the ants walking on the trails (Supplementary Fig. S1). An additional camera was placed on the stake, looking sideways at the ants, to allow another perspective for behavioral analysis. Filming lasted from 19:30 to 00:00 for 4–7 nights for each trail (Supplementary Table S2). Timing of filming was based on previous work showing activity begins around 19:30 and peaks around 21:00^15^. Infrared lights (IR30, CMVision, Houston, USA) were connected 12-Volt 7Ah batteries (UP1270, UniPower, São Paulo, Brazil) to allow illumination of the trail without disturbing the behavior of the ants. The camera batteries lasted for approximately 1.5 hours, so the battery was changed once in the middle of a filming period. Slight adjustments in where the trail was positioned in the video view would sometimes occur at this time. Figure 1a shows an example image of a trail filmed and images of the remaining trails filmed are found in Supplementary Fig. S2.

### Automated ant tracking

A total of 78 hours and 56 minutes of video were recorded for seven colonies across four nights (Supplementary Table S2). We developed a machine learning approach to process and analyze these videos using a deep learning based segmentation model that identified ants as they came onto the screen and tracked them as they moved across the screen (Supplementary Material).

Our automatic ant tracking method contains two main processes: (1) detecting ants in each image frame of all videos, and (2) building ant trajectories for every video based on the detected ants. Commonly, deep learning schemes require a large amount of labeled ground truth data for model training. Since our dataset is quite large (>8 million image frames), we aimed to generate sufficient labeled data for training our deep learning model without incurring excessive human labeling effort. Also due to the large size of our dataset, common active learning based sample selection methods (e.g.^21^) are not efficient. The goal of ant detection is to build ant movement trajectories and since ant trajectories normally span multiple consecutive frames in videos, detected ant positions in earlier frames assist with ant detection in later consecutive frames. That is, while ant detection forms a basis for building ant trajectories, trajectories of detected ants may also help ant detection. Hence, we designed our trajectory building procedure such that it not only can track detected ants but also can provide cues to indicate where (which frames and locations) there might be inconsistencies in ant trajectories and difficult scenarios for ant detection (e.g. densely clustered ants). We used such cues to select difficult cases from the frames for labeling to improve the deep learning detection model as well as the ant detection results. Therefore, our detection-tracking method consists of two rounds (with the second round improving the detection and tracking results of the first round), and each round performs two major steps, ant detection and trajectory building, as described below.

#### Ant detection

This aims to detect ants in all the frames of the videos. We applied a novel object detection and segmentation model, Mask R-CNN^22^, to automatically detect ants in every frame.

#### Ant trajectory building

Given the detected ants in each frame, the next step is to form ant trajectories that connect detected ants frame-by-frame in videos. We formulated this ant trajectory building problem as a *transportation problem*, that is, between every two consecutive frames in each video, we find an optimal transportation (for ants) that corresponds to real movement of ants. In this transportation formulation, each detected ant in frame *K* can be viewed as a ‘supplier’ and each detected ant in frame *K* + *1* can be viewed as a ‘receiver’. The dissimilarity (based on spatial distance and appearance difference) between ants in two consecutive frames is a measure of how much ‘cost’ it would take to transport (move) one ant in frame *K* to another in frame *K* + *1*. The objective is to transport detected ants (as many as possible) in frame *K* to frame *K* + *1* with the minimum total cost. Optimal transportation based tracking methods are known to be effective for tracking sets of moving and changing objects in image sequences^23,24^.

In the first round, we randomly selected frames to label as training data. This allowed us to quickly and unbiasedly obtain data samples for training a decent detection model. We then applied the trained model to all of the frames to produce ant detection results. We conducted trajectory building on detected ants to form the ant trajectories. Besides tracking ant movement, our trajectory building procedure in the first round also provided cues for identifying inconsistencies in ant trajectories and difficult cases in the frames for ant detection. In the second round, we applied training data selection to those difficult cases to find additional frames for labeling, and the enlarged training dataset thus obtained was used to re-train the Mask R-CNN detection model. The re-trained detection model was then applied to all the frames to produce the final ant detection results, which were used to build the final ant trajectories in the videos.

To identify difficult cases for additional training data selection, we used the following set of measures to capture possible errors in ant detection and trajectory results. (i) Ant speed: At a place where ants usually do not move very fast but a fast movement is suggested by the optimal transportation solution, this instance might indicate an error in ant detection. (ii) Missing ants in the middle part of a tree branch: When the optimal transportation solution does not find a corresponding ant instance in the next frame in the interior section of a tree branch, it might suggest a missing data point in ant detection. (iii) Ant identification (ID) switching: Each detected ant was assigned an ID number; when multiple ants are seen at spatially close interaction and slight changes on the dissimilarity scores among these ants give largely different solutions for the optimal transportation problem, this might suggest an ant ID switch error. Based on these observations and measures, our trajectory building process can help identify difficult detection and tracking cases for additional training data selection to improve model performance.

Overall, we annotated 20,666 images for training the deep learning model for the ant detection task. Thus, the model is fairly robust to complex backgrounds, low contrast image areas, illumination differences. Besides relying on the training data and the robustness of Mask-RCNN model, our tracking algorithm works on the temporal information and is also robust to false-detection and miss-detection of ant. In particular, our tracking algorithm is tuned to be very robust to false-positive detections. Namely, our tracking algorithm has a strong prior/preference to discarding false-detections using temporal information. When we train and apply the Mask-RCNN model, we tolerate the Mask-RCNN model to produce some false-positive detections in order to keep the number of miss detections very low. For occasional miss-detection cases, our tracking algorithm can also recover them using temporal information.

Our automatic ant detection and tracking method extracted the *x* and *y* coordinates in pixels of detected ants in every frame and assigned each ant an identification number (Fig. 1a and Supplementary Video S1). Ant identification numbers were used to form ant trajectories used in further analysis.

### Error assessment

To assess the accuracy of the computer model, we watched a subset of videos and determined the error rate. GoPro cameras automatically divide footage into 26-minute-long videos, so one night of footage at a single trail has 6 to 10 videos. This provides a way of checking the accuracy of the computer tracking at random points throughout a night. We first error checked videos from the middle of the night (when the trails should be busiest) to determine if the data from that colony was high enough quality to use in our analysis. If the average accuracy was greater than 60% for these videos, we continued to error check all videos and nights for that colony. To error check, we counted the number of ant trajectories with errors out of the first 15–30 tracked ants. The number of ant trajectories checked varied because videos from early in the foraging period sometimes had fewer ants.

To ensure consistency in the type of ant trajectories that were analyzed, trajectories beginning in the middle of the field of video view were removed. This created uniformity between all colonies and nights in the type of ants that were compared as it focused on the ants that made it from one end of the trail to the other completely in the view of the video.

### Trajectory analysis

We used R version 3.4.4 and RStudio version 1.1.447 for all analyses^25,26^. Ant location data was frame-by-frame, so we used the native frame rate of the cameras (29.97 or 25 frames per second; the default setting of the cameras varied) to convert the time in frames to seconds and then used the start times of each video to convert it to real time (Supplementary Table S2). To convert ant location data from pixels to centimeters, we placed a ruler in each video to determine the conversion factor (Supplementary Fig. 2).

To determine how individual ants were moving, we calculated the following variables: average speed, overall direction, and straightness. Average speed was taken as the total distance an ant travels while in the video over the time it takes for them to travel that distance. Overall direction was whether the ant headed away from or towards the nest which we determined based on where the ant entered and exited the video view. A variety of measures are used to determine the straightness or tortuosity of an animal’s movement path^27,28^. Ant movement on trunk trails is expected to move in an oriented direction, and not be a random search path, thus we used the simplest measure, the straightness index^28^. The straightness index (ST) is a ratio between the net displacement and total path length:

$${\rm{ST}}={\rm{d}}/{\rm{L}};$$

where d = the distance between the beginning and end of the path and L = total path length.

To assess similarity between individual ant trajectories, we calculated an exploration index (EI) for each trajectory (Supplementary Information). The exploration index measures how much an individual trajectory covers unique areas of the trail space. First, we computed an Ants Visiting Map (Supplementary Information) for a video which estimates how frequently ants are visiting different parts of the trail. We then scored grid cells of the trail space based on how many trajectories pass through each cell. The exploration index for an individual trajectory is calculated from the scores of the grid cells that the trajectory passes through. If a trajectory mostly passes through areas of the trail space that are visited by many ants, the individual trajectory will have a low EI. To control for trajectory length, we divided the EI for a trajectory by trajectory length to get an average exploration index (AEI) for each trajectory.

Inspection of the trajectories showed that some ants performed U-turns, where they would exit the field of view from the same side that they entered on (Supplementary Video S2). To more accurately represent the shape of the trajectories, we broke U-turning trajectories into two parts at the point the trajectory turned from one direction on the trail to the other and calculated straightness and exploration for the different trajectory parts individually.

### Statistical analysis

A linear mixed-effects model was used to assess whether the speed of ants changes over a foraging period. The model was generated using the lmer function in the R package’ lme4’^29^, with speed as the response variable, time as the fixed effect, and colony and date as the random effects. The package ‘lmerTest’^30^ was used to generate p-values. We checked the plotted residuals to ensure homoscedasticity prior to utilizing the results of the model. We also used a linear mixed-effects model to test whether the trajectories of ants with lower straightness scores have higher exploration values. We included colony, date, and video as random effects. We fit our model with the straightness index (ST) as the fixed effect and the response variable as the average exploration index (AEI).

To analyze whether exploration differed across a foraging period, we compared the average exploration index within 30-minute intervals across the recording period. We pooled our data within 30-minute intervals to overcome discrepancies in recording times across dates. We fit a linear mixed-effects model with the interval as the fixed effect, colony and date as random effects, and the AEI for that interval as the response variable. We used a comparison of means with the Tukey method to investigate how the AEI of trajectories differed between 30-minute intervals.

## Results

### Automated tracking performance

The automated tracking of ants in video frames resulted in 20,230,585 data points on ant movement. The model had two types of accuracy against which it can be judged, relative to a human. The first is species accuracy (detection accuracy) which is a measure of how well the model recognized the correct species of ant. The model correctly detected *C*. *rufipes* ants with an accuracy of 97.86%. The model picked up other insects or species of ants on the trail (false positive) or failed to detect a *C*. *rufipes* ant as it went across the trail 2.14% of the time.

The second accuracy measurement is tracking accuracy. The computer had to detect *C*. *rufipes* ants and follow them as they moved across the screen. If an ant moved in a straight line this required the computer to recognize and track that ant for about 4 seconds or 120 frames. The computer assigned identification numbers to individual ants to follow an ant as it travelled across the screen. The machine learning model sometimes made errors in doing this. The computer may switch identification numbers when ants walked too closely together (Supplementary Video S3). An average of 78.70% of complete ant trajectories across all colonies had no mistakes as identified by a human observer (Supplementary Table S3). The tracking accuracy was the lowest for colonies MP2 (40.0%), MP11 (31.7%), and MP17 (50.6%). Identification number switches commonly happened in colonies MP2 and MP11. These trails were very thin and introduced more challenges in determining the trajectories of individual ants, so they were removed from further analysis. We have additionally removed colony MP17 as an obstruction in the trail led to ants departing from the branch and walking underneath leaves (Supplementary Video S4). Ants disappearing under leaf debris made it difficult to track an individual ant. We have made all videos and data available as we expect improved future machine learning models can make use of them.

The exclusion of these colonies brought the size of the dataset to 8,412,477 data points on ant movement from four colonies: MP1, MP6, MP10, and MP16. The large reduction in number of data points from the elimination of 3 colonies can be attributed to the configuration of these trails creating congested areas on the trails where single ants were tracked multiple times falsely inflating the number of ants and overall data points. The data points from the 4 included colonies represents the movement data for 64,498 ants. The average tracking accuracy of the remaining colonies was 81.39% (MP1: 72.0%; MP6: 82.1%; MP10: 77.2%; MP16: 92.1%). Most errors were due to an identification number switching to a different ant (8.28%). The high error rate for colony MP1 could be attributed to the darkness of the videos causing the model to miss part of an ant’s trajectory or failing to detect an ant in the dark areas of the trail. If we consider only the errors where a number is on a wrong ant or a number is not on an ant, the accuracy improves greatly (overall: 90.94%; MP1: 91.5%; MP6: 88.8%; MP10: 86.6%; MP16: 96.3%). We are mainly concerned with the direction and shape of trajectories, and the main error that impacts an individual ant’s trajectory is when ants switch to the wrong identification number, so the second calculation of accuracy rate is more reflective of this.

### Collective movement pattern

Most ants walk on the same area of the available trail space (Fig. 1). The trail usage pattern is consistent between nights (Fig. 1c). The mean speed of all ants from all colonies and nights was 5.15 cm/s ± 1.63 (standard deviation). The average speed of the colonies ranged from 4.74 cm/s to 5.62 cm/s and within colony variability in speed was similar between colonies (mean (cm/s) ± standard deviation; MP1: 4.94 ± 1.72; MP6: 5.58 ± 1.62; MP10: 4.82 ± 1.55; MP16: 4.72 ± 1.43). The results of the linear mixed effects model showed that ant speed decreases by 0.45 cm/s ± 0.07 (standard error) throughout the night (t_(94)_ = −6.60, p < 0.0001) (Supplementary Fig. S3).

### Individual trajectory analysis

**Figure 2:**
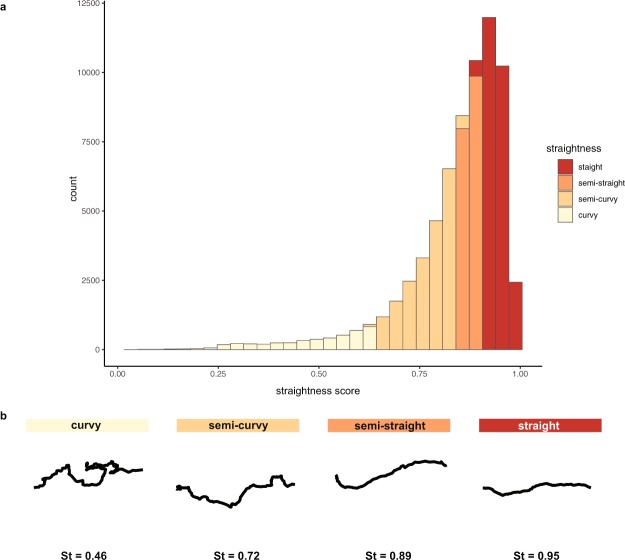
Straightness of trajectories. (**a**) Histogram showing the distribution of straightness scores of ant trajectories for all nights and colonies. (**b**) Example trajectories for ants with different straightness scores. The straightness score (St) for each trajectory is included below. All 4 example trajectories were taken from the same colony and night (colony MP16 – January 15). Supplementary Video S5 features video of the example ants with their trajectories annotated.

Most ants walked in nearly straight lines (Fig. 2a). However, the negative skew of the distribution highlights the tendency of ants with low straightness scores to wander across the trail (Fig. 2a,b and Supplementary Video S5). The median straightness score across all colonies was 0.88 and was similar for each colony (MP1: 0.87; MP6: 0.89; MP10: 0.86; MP16: 0.87). We fit a beta mixture model using the R package betareg^31^ to determine whether the distribution represents different groups. We used the Bayesian Information Criterion (BIC) to assess model fit and found that the distribution was best represented by four groups: straight (37.0%; n = 25,224; mean straightness = 0.94), semi-straight (26.2%; n = 17,840; mean straightness = 0.88), semi-curvy (30.0%; n = 20,437; mean straightness = 0.77), and curvy (6.8%; n = 4623; mean straightness = 0.49). The semi-curvy straightness group has a minimum straightness score of 0.64, so 93.2% of ants have straightness scores greater than 0.64.

**Figure 3:**
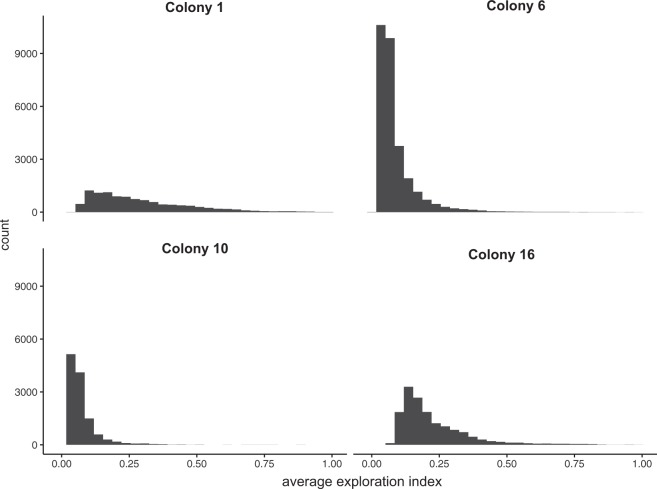
Average exploration of trajectories for different colonies. Histogram showing the distribution of the average exploration index values for all trajectories divided by colony. The average exploration index varies from 0 to 1, with 1 indicating the highest amount of exploration.

**Figure 4:**
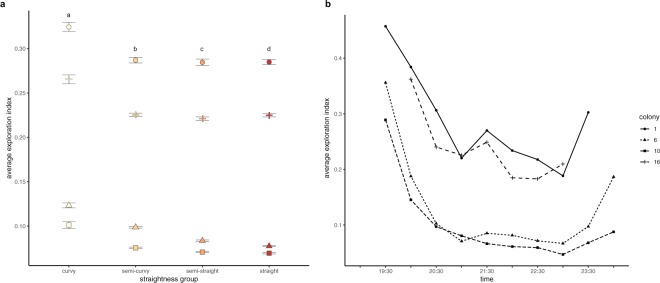
Average exploration across time and for different straightness groups. (**a**) The mean of the average exploration scores for the trajectories in each of the straightness groups from Fig. 2a. Lines indicate ± standard error of the mean. Superscripts indicate straightness groups as significantly different (linear mixed-model p < 0.0001). (**b**) The mean of the average exploration scores for all trajectories within each 30-minute interval across the recording period, divided by colony.

The distributions of average exploration index (AEI) of trajectories differed in shape for each colony (Fig. 3). Across all colonies, a majority of ants showed low levels of exploration, but the positive skew of the AEI distributions indicates a group of ants that are more exploratory (Fig. 3). Colony 1 had the highest median AEI at 0.24, closely followed by colony 16 at 0.19. The median AEI for colony 6 and colony 10 were both approximately 0.06. There was a weak negative relationship between the straightness of a trajectory and its exploration value, as average exploration was estimated to decrease by 0.11 ± 3.14e-3 as straightness increases (linear mixed-effect model; t_(6810)_ = −36.09, p < 2e-16). The straightness groups significantly differed in average exploration (Fig. 4a; linear mixed-effect model; t_(6810)_ = −11.03, p < 2e-16). Post-hoc analysis using the Tukey Test showed that ants with curvy trajectories had the highest AEI followed by ants with semi-curvy trajectories, then ants with semi-straight trajectories, and ants with straight trajectories had the lowest AEI (linear mixed-effect model; Tukey Test; p < 0.0001).

### Temporal pattern

Average exploration of trajectories decreased from the beginning of the foraging period to the middle of the foraging period, before increasing slightly again (Fig. 4b). The AEI was significantly greater (linear mixed-effect model; Tukey Test; p < 0.0001) at the beginning of the night to all other time intervals. However, the AEI at 22:30 was significantly lower (linear mixed-effect model; Tukey Test; p < 0.0001) than at 23:30 or 00:00.

## Discussion

Our study used an unobtrusive filming set-up to record behavioral data on more than 64,000 ants moving in a rainforest at night in an area of high disease pressure. Most ants walk in a straight line across the trail, matching our prediction of how ants might behave when using trunk trails (Fig. 2). Similar to straightness, most ants show low levels of exploration, but a subset of ants cover unique areas of the trail (Fig. 3). Average exploration of ants was higher at the beginning of the foraging period (Fig. 4b). Exploration may enhance food discovery, but the low levels of exploration exhibited by the majority of ants may protect most foragers from the risks associated with venturing from the main trail.

The variation in exploration of trajectories indicates that the ants may have different foraging roles. Social insects have members of the colony known as scouts that assist in discovering and recruiting the colony to new food sources^32–35^. The higher exploration levels at the beginning of the night indicate that perhaps some of those ants are acting as scouts and recruiting ants to new food sources. Recruits should subsequently show lower levels of exploration than the scouts as they follow a pheromone trail to the food source. Forager categories can extend beyond just scouts and recruits, as a forager’s experience level and information source will alter its behavior^36,37^. A forager recently recruited to a food source must engage in some searching behavior as they follow external stimuli to the food source. Meanwhile, a forager that has already made the trip to a food source is familiar with the route and should exhibit less searching behavior. Considering the variation in forager information may explain the distributions of exploration and straightness scores, showing all different levels of straightness and exploration.

A majority of the trajectories likely represent ‘employed foragers’^38^, or foragers repeatedly exploiting a known resource, since the trails last for multiple months and usually visit a stable homopteran or honeydew secretion. Employed foragers should have lower exploration scores, as their trajectories will overlap other trajectories and this has implications for disease risk. Fungal infected cadavers surround the trunk trails of *Camponotus rufipes* in this habitat, likely dropping spores directly onto the trails below^17^. It is not possible to quantify the abundance and distribution of micron sized spores on trails in a forest, but the long term tracking of cadaver abundance and the proximity to the trails implies spore presence on the foraging trails^17^. Thus, for most ants, only the first ants walking across the trail after spores have dropped would likely pick up spores. In contrast, ants with higher exploration scores, the “explorers”, are constantly more likely to encounter a spore that has not been picked up by a different ant. Through the same logic, an explorative ant has a higher chance of discovering a new food source, demonstrating the benefits of this searching behavior.

We filmed only a small area of the foraging trails, providing a brief snapshot of an ant’s behavior. To know whether higher exploration values represented ants that were more likely to wander from the trail and discover new food sources, one would need to follow individual ants for their entire foraging trip, which was beyond the scope of this study. In our study area, exploration values were also impacted by the size of the trail, as ants will have higher overlap (= lower exploration) on narrower trails. The wider trails (colony 1 = 6 cm and colony 16 = 7 cm) had higher median exploration scores than the narrower trails (colony 6 = 3 cm and colony 10 = 1.7 cm). Observing ants beyond one portion of the trunk trails could remove differences between colonies on exploration based on trail size. Trail width still has implications in the context of disease exposure, however, as wider trails offer more substrate for possible spores and perhaps colonies that use larger trails have higher levels of infection.

Following individual ants for their entire foraging trip would also clarify whether individual ants vary in their level of exploration across a foraging trip. Experienced foragers tend to continue exploiting the same food source until it runs out^33^. Moreover, individual ants have been shown to be consistent in their exploratory behavior^39^. The ants with low exploration values appear to be in retrieval mode and thus will likely continue exhibiting the same levels of exploration. Laboratory studies on trail bifurcations provide some evidence on the likelihood of ants to explore away from the main trail. For example, when Argentine ants (*Linepithema humile*) were placed in a maze to a food source, over 80% of the total traffic used the shorter path to the food source in the majority of experiments^40^. Ants selecting a longer path, and ignoring pheromone signals, could represent patrollers or explorers. In a study on Pharaoh’s ants (*Monomorium pharaonis*), 30% of the foragers failed to reorient themselves when placed into a trail network without other ants^41^. Perhaps these ants that fail to correctly follow the trail represent another group of foragers and match up with the exploratory group observed in our study.

Beyond food discovery and retrieval, other species of ants provide evidence of more roles within foragers, such as trail maintenance and defense. Ants were observed carrying leaves (Supplementary Video S6), although this could be for nest material and not trail cleaning. Another role could be maintaining the pheromone trail. For example, *Atta sexdens* minims help with the pheromone trail instead of food transport^42^. Ants were observed dragging their gaster on the trail likely depositing trail pheromone (Supplementary Video S7). U-turning ants have been shown to deposit pheromones at a higher rate^43^. Perhaps the main distinction between the groups is not in trail exploration, but in pheromone deposition, with the U-turners serving as the ants that are maintaining the strong chemical signal and allowing most ants to walk directly across the trail.

The different walking styles could also reflect defensive behavior. Smaller workers hitchhike on leaf fragments carried by larger workers in *Atta colombica* leaf-cutting ants, and this likely serves as a defense against parasitoid Phorid flies^44^. Flies, that could possibly be parasitoids, were observed closely following ants on the trail and in some cases appearing to land on the ants which may indicate laying eggs which later become endoparasitoids (Supplementary Video S8). Although the prevalence of parasitoid flies attacking *C*. *rufipes* is unknown, we have observed adult ants infected by decapitating phorid flies in our study area (Supplementary Video S9). The presence of phorids could directly cause the exploring and U-turning behavior, as ants attempt to avoid flies landing on them. A follow up study could investigate this question of parasite avoidance by directly quantifying how ants behave when phorids are in the environment.

In this study, however, we focused on variability in individual forager trajectories. We found a group of foragers that explores more areas of the trail. Increased exploration increases a forager’s chance of encountering a new food resource while simultaneously increasing their exposure to possible risks. The variability in forager behavior provides a possible mechanism for how a colony might mitigate risk through only having a small percentage of foragers exploring out from the safety of the main trail. The scale of our dataset, and ability to collect this data across multiple nights and colonies, increases the reliability and strength of our conclusions. Combining computational advances with behavioral observations provides a technique to investigate the mechanisms of individual movement patterns that influence the distribution of animals in time and space.

## Supplementary information

Supplementary Information and Figures

Supplementary Video S1

Supplementary Video S2

Supplementary Video S3

Supplementary Video S4

Supplementary Video S5

Supplementary Video S6

Supplementary Video S7

Supplementary Video S8

Supplementary Video S9

Supplementary Table S1

Supplementary Table S2

Supplementary Table S3

## Data Availability

The original videos analyzed in this study, along with the full tracking dataset, are accessible through Pennsylvania State University’s institutional repository ScholarSphere (10.26207/q14b-gx36). Information about our process of analyzing the videos and links to the code used can be found in the Supplementary Information.
