# Supplementary material for: Automated tracking and analysis of ant trajectories shows variation in forager exploration
Source: Sci Rep. 2019 Sep 13;9:13246. doi: 10.1038/s41598-019-49655-3 (PMC6744467; doi:10.1038/s41598-019-49655-3)
Supplement: Supplementary file 1 — Supplementary Information and Figures [file 41598_2019_49655_MOESM1_ESM.docx]

**Supplementary material for:**

**Automated tracking and analysis of ant trajectories shows variation in forager exploration**

Natalie Imirzian, Yizhe Zhang, Christoph Kurze, Raquel G. Loreto, Danny Z. Chen, & David P. Hughes

1. **Description of our ant detection and tracking approach**

The overall pipeline of our approach for ant detection and tracking is sketched in the following figure. We first apply a Mask R-CNN model [2] on every image frame in a video to detect all ants (and their positions) in the frame; we then apply an optimal transportation based tracking method [1] to match and connect the detected ants in each frame to form ant trajectories for individual ants throughout the video.


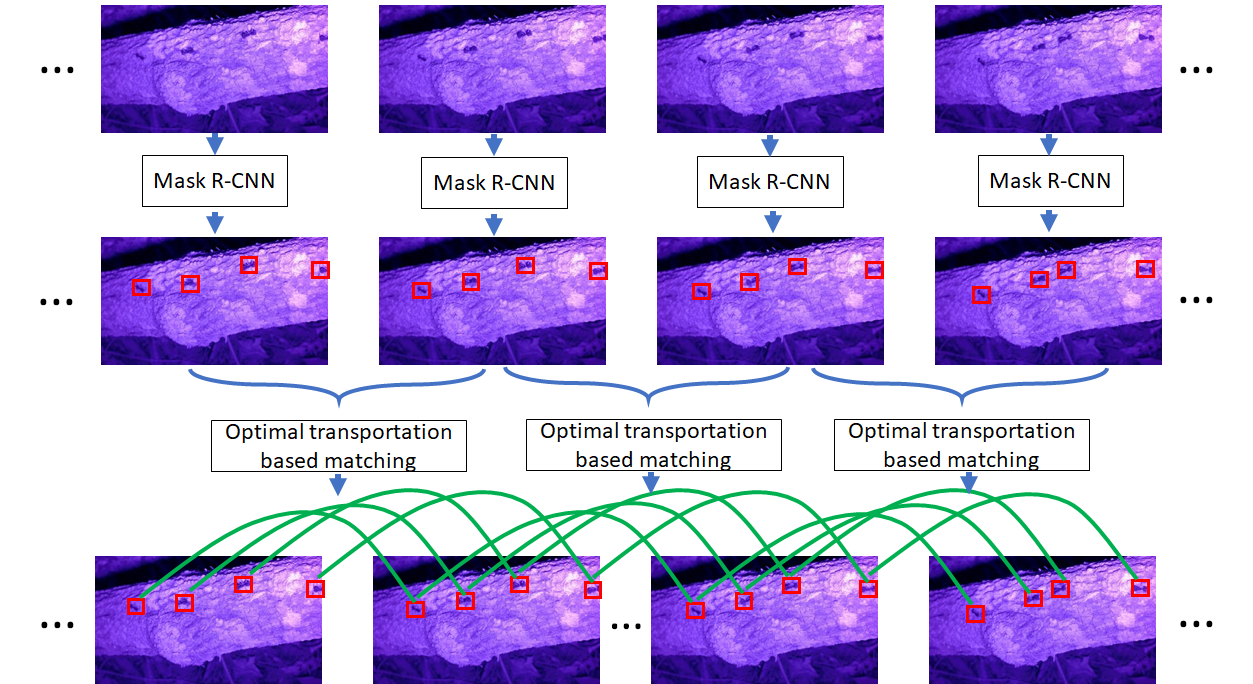


*The Mask R-CNN model:*

Overall, we hand-labeled 20666 images for training a Mask R-CNN model [2]. Our code for training and testing the Mask R-CNN model can be found via the Google Drive link below:

https://drive.google.com/drive/folders/15w3VAhG9vvc0-Psx2cjoEpBh8EFy4rTE?usp=sharing

*Optimal transportation based ant matching/tracking method:*

We applied the tracking method developed in [1] for tracking ants detected by the Mask R-CNN model. Our code for this method can be found via the Google Drive link below:

https://drive.google.com/drive/folders/19REUh1HmD97niFB4KlkZ8QxeqR6dRW6h?usp=sharing

[1] J. Chen, C. W. Harvey, M. Alber, and D. Z. Chen. A matching model based on earth mover’s distance for tracking Myxococcus xanthus. In *International Conference on Medical Image Computing and Computer-Assisted Intervention (MICCAI)*, pp. 113-120, 2014.

[2] K. He, G. Gkioxari, P. Dollar, and R. Girshick. Mask R-CNN. *IEEE International Conference on Computer Vision (ICCV)*, pp. 2980-2988, 2017.

1. **Ants Visiting Map**

Trajectories can be embedded into the 2D space of the images in the video, as follows. Suppose we divide an image into a grid structure of size *m x n* (i.e., the image consists of *m x n* grid cells). Then we can represent an ant trajectory *T* as a sequence of grid cells that *T* travels or visits. Further, for each grid cell *C(i, j),* we can count the number *V(i, j)* of times that *C(i, j)* is visited by the ant trajectories. This will generate a map of the image which has a similar effect as a heat map: The larger the value *V(i, j)* is (i.e., the more often the cell *C(i, j)* is visited by the ant trajectories), a higher temperature the cell *C(i, j)* has.

Suppose we have *K* trajectories *T_1_, T_2_, . . . , T_K_.* For a trajectory *T_k_*, it consists of a sequence of grid cells *C(x^k^_1_, y^k^_1_), C(x^k^_2_, y^k^_2_),* . . . , *C(x^k^_Pk_, y^k^_Pk_),* where *P_k_* is the number of cells in trajectory *T_k_*.

We compute the “ants visiting map" of trajectories using the following procedure.


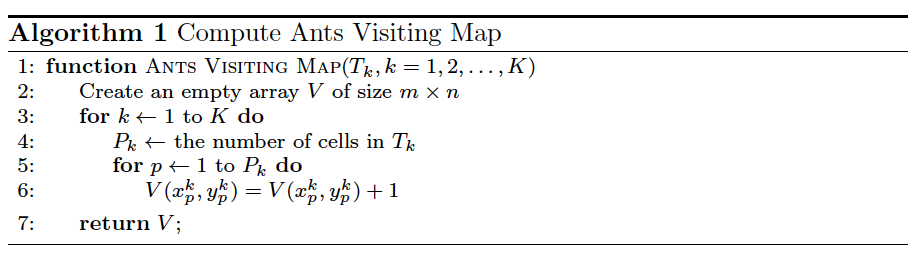


The following is an example image output from the above calculation:

**
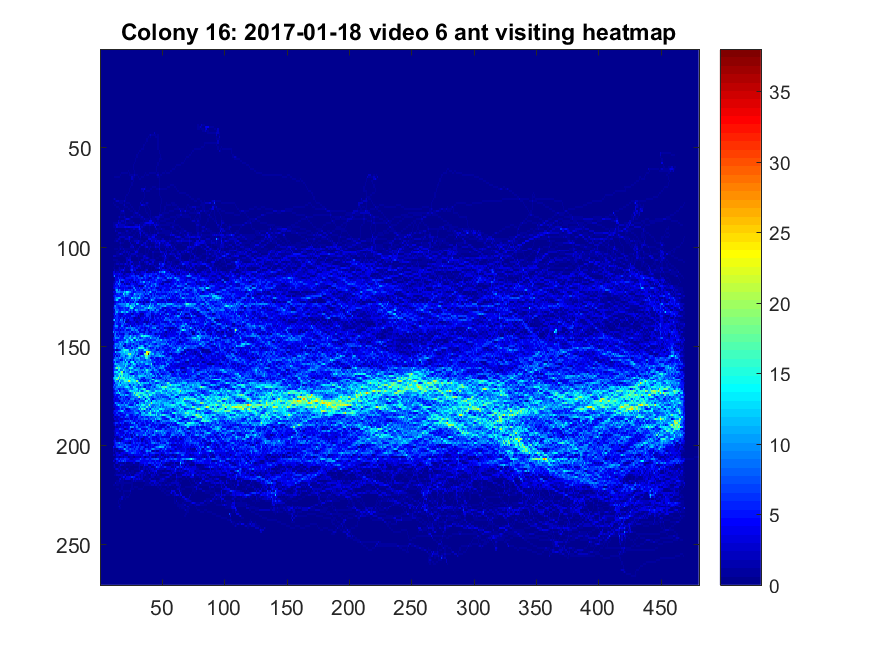
**

1. **Exploration Index**

After computing the ants visit map, we can further compute an index, called the exploration index (EI), of each trajectory.

The exploration index may better capture the ant exploration of the image areas. For every grid cell *C(i,j)*, we give it an initial exploration value *E(i,j)*. To avoid introducing any bias here, we set *E(I,j)* as a constant (e.g.,1) for every grid cell *C(i,j)*. Then when a trajectory *T* passes through cell *C(i,j)*, it picks up a value *E(i,j)/V(i,j)*, where *V(i,j)* is the number of trajectories that visit *C(i,j)* computed in the above procedure. That is, the more trajectories pass through cell *C(i,j)*, a lower value of exploration that the trajectories will pick up from visiting *C(i,j)* (as *C(i,j)* is visited by multiple ants, its exploration value becomes low. We may then compute the total sum of exploration values in all the cells of trajectory *T* as the exploration index (EI) of *T,* and also the average exploration index (AEI) of *T* which can be obtained by dividing the EI by the length of the trajectory. When comparing between videos in a night of footage, we removed short videos or early videos with only a few trajectories as this gives inaccurate exploration indices.

**
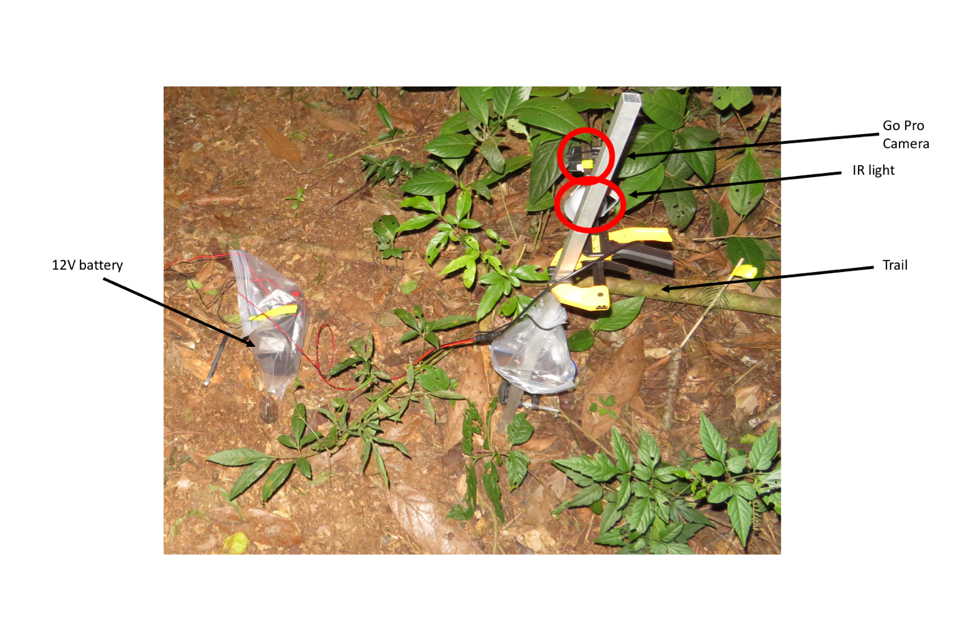
**

**Supplementary Figure S1.** Image of camera set-up. GoPros were attached to a 30cm clamp that was attached to a pole placed in a ground 30cm from the trail. An infrared light was attached the end of the clamp next to the GoPro and powered by a 12-volt battery (pictured to the left). The camera was approximately 30 cm above the trail.

**Supplementary Figure S2.** Images of trunk trails filmed for all colonies. Images taken from GoPro footage, pink/purple color due to infrared light and infrared filter in camera.


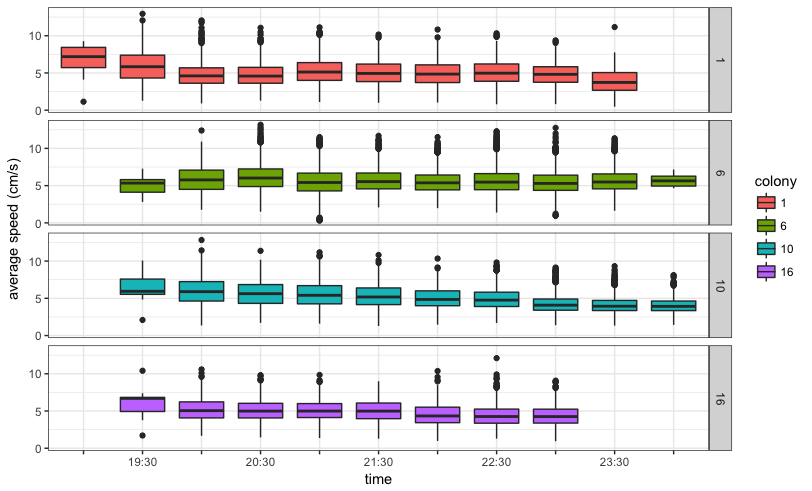


**Supplementary Figure S3. Speed of ants over time.** Average speed of ants within a 30-minute interval, broken down by colony.
